# Protocol to use de-epithelialized porcine urinary bladder as a tissue scaffold for propagation of pancreatic cells

**DOI:** 10.1016/j.xpro.2022.101869

**Published:** 2022-11-21

**Authors:** Michael Karl Melzer, Markus Breunig, Paul Lopatta, Meike Hohwieler, Sarah Merz, Anca Azoitei, Cagatay Günes, Christian Bolenz, Alexander Kleger

**Affiliations:** 1 Clinic of Urology, Ulm University Hospital, 89081 Ulm, Germany; 2 Institute for Molecular Oncology and Stem Cell Biology, Ulm University Hospital, 89081 Ulm, Germany; 3 Clinic of Internal Medicine I, Ulm University Hospital, 89081 Ulm, Germany; 4 Core Facility Organoids, Ulm University, 89081 Ulm, Germany

**Keywords:** Cell culture, Stem Cells, Cell Differentiation, Tissue Engineering, Material sciences

## Abstract

*Ex vivo* organ culture can be a useful alternative to *in vivo* models, which can be time-, labor-, and cost-intensive. Here we describe a step-by-step protocol to use de-epithelialized porcine urinary bladders as scaffolds in air-liquid interface *in vitro* culture systems for a variety of pluripotent stem-cell-derived and patient-derived pancreatic cells and organoids. The scaffold can trigger cell maturation and enable cell-cell interaction and invasion capacity studies. However, this model is limited by the lack of functional vasculature.

For complete details on the use and execution of this protocol, please refer to Melzer et al. (2022),^1^ Breunig et al. (2021),^2^ and Breunig et al. (2021).^3^

## Highlights

• Step-by-step protocol to enable porcine urinary bladder organ culture
• Easy and time-resolved access to *ex vivo* cultures with *in vivo* features
• Robust histology-based readout of *ex vivo* propagated cell types
• Successful modelling of early and late steps during pancreatic carcinogenesis

## Abstract (editor highlights)

Publisher’s note: Undertaking any experimental protocol requires adherence to local institutional guidelines for laboratory safety and ethics.

## Graphical abstract

**Figure:**
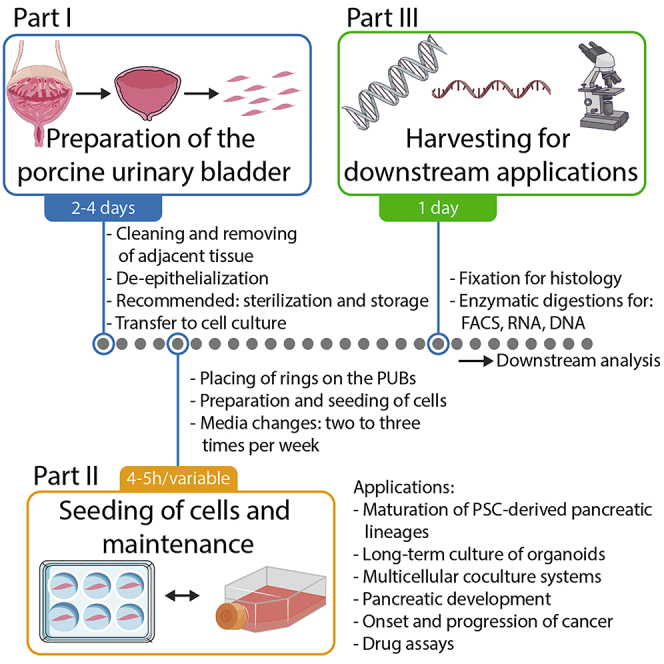

## Before you begin

The protocol below describes the specific steps to employ a porcine urinary bladder (PUB) as an organ culture model for different pancreatic cell types. The protocol was initially established using human embryonic stem cell (hESC)-derived pancreatic progenitor cells (PPs) and pancreatic duct-like organoids (PDLOs). In a next step, we have successfully implemented the PUB *ex vivo* model to investigate human pancreatic cancer patient-derived organoids (PDOs), pancreatic stellate cells, and pancreatic cancer cell lines.

The *in vitro* differentiation of human pluripotent stem cells (hPSC) into PPs and PDLOs^2^^,^^3^ as well as propagation and maintenance of PDOs^4^^,^^5^ has been described in detail (e.g., step-by-step protocols^3^^,^^6^). Pancreatic stellate cells and cancer cell lines were cultured using standard media conditions.^7^^,^^8^

### Institutional permissions

All experiments were performed in accordance with the institutional and governmental regulations of Ulm University and the state of Baden-Württemberg in Germany. PUBs were obtained as commercial byproduct of the food industry and thus, did not need further specific approval. However, such approval may be necessary in certain countries and should be obtained before starting the procedure.

Culture and differentiation of hESCs toward the pancreatic lineage were performed with permission from the Robert Koch Institute according to the “79. Genehmigung nach dem Stammzellgesetz, AZ 3.04.02/0084” in Germany. PDOs were propagated from patients after obtaining written informed consent and in compliance with institutional approval by the local ethics committee of Ulm University.

Please ensure that all legal and ethical requirements to culture and differentiate human embryonic stem cells and to isolate patient-derived organoids are met before beginning with the protocol.

### Preparation of solutions

**Timing: 3 h- variable**

1. Bladder wash solution (3% antibiotic-antimycotic solution in DPBS).
  a. Prepare 485 mL of DPBS by removing 15 mL of a 500 mL bottle with sterile DPBS.
  b. Add 15 mL of antibiotic-antimycotic solution to obtain a 3% solution (equal to 300 IU/mL penicillin, 300 μg/mL streptomycin, 750 ng/mL amphotericin B).
  c. Store at 4°C for up to 3 months.
2. 10 mM sodium acetate and 5 mM calcium acetate solution for preparing dispase-II stock solutions.
  a. Dissolve 820.3 mg sodium acetate (molecular weight 82.03 g/mol) and 790.85 mg calcium acetate hydrate (molecular weight 158.17 g/mol) in 800 mL dH_2_O.
  b. Mix well and carefully adjust pH to 7.5 by adding 1 M sodium hydroxide (NaOH) if pH is under 7.5 or alternatively 37% hydrochloric acid (HCl) if pH is above 7.5 dropwise to the solution.
  c. Fill up to 1 L with dH_2_O.
  d. Store at room temperature (usually 18°C–22°C) for up to 1 year.

***Note:*** Storage for a longer period of time may be possible but was not tested.

3. Dispase-II solution (stock solution 100 mg/mL, working solution 10 mg/mL).
  a. Add 10 mL of 10 mM sodium acetate and 5 mM calcium acetate to 1 g of dispase-II powder.
  b. Dissolve dispase-II powder by pipetting up and down for around 3 min.
  c. Filter dispase-II solution (100 mg/mL) sterile with a 0.22-μm filter.
  d. Store the 100 mg/mL dispase-II stock solution for up to 3 months at 4°C.
  e. Dilute the stock solution in a ratio of 1:10 to create a 10 mg/mL working solution in sterile DPBS **with** MgCl_2_ and CaCl_2_.
  f. Always prepare working solutions freshly.
4. 2% agarose in PBS.
  a. Add 2 g of agarose powder to a beaker.
  b. Add PBS and dissolve agarose solution by heating in a microwave.
  c. Fill up to 100 mL PBS.
  d. Autoclave (120°C 15 min) 2% agarose solution.
  e. Store at 4°C for up to 3 months.
5. Sterilization solution for PUBs (0.1% peroxy-acetic-acid (PAA) in PBS)
  a. Prepare a bottle with 349 mL sterile DPBS.
  b. Add 1 mL of 35% PAA.
  c. Mix by slight swirling.
  d. Always prepare freshly.

**CRITICAL:** Handle PAA very carefully as it is a hazardous reagent. Follow the manufacturer`s indications on how to handle and store it. Wear personal protection equipment (gloves, clothing, eye, and face protection) and store cool at 4°C.

6. GFR-Matrigel.
  a. To thaw original Matrigel vials, place them on ice and transfer the container with the ice into a 4°C–8°C environment for 10–16 h.
  b. Pre-cool tips and reaction tubes at −20°C before handling the Matrigel.
  c. Prepare 1 mL aliquots and store at −20°C for up to 2 years (check certificate of analysis for each batch).
  d. Thaw the required number of aliquots on ice for 30 min to 1 h before use.
  e. Avoid repeated freeze-thaw cycles of aliquoted Matrigel.

***Note:*** In this protocol, we have used Matrigel with a protein concentration ranging from 8.8 to 11.3 mg/mL and an endotoxin content of less than 1.5 EU/mL.

7. ROCK inhibitor Y-27632 (10 mM).
  a. Volumes are calculated for a molecular weight of 338.28 g/mol.
  b. Original vials must be centrifuged before opening.
  c. Dissolve 10 mg in 2.956 mL of sterile, double-distilled water by pipetting.
  d. Store 100 μL aliquots at −20°C for no more than 6 months.

***Note:*** Specific molecular weight may be variable between batches. Therefore, volumes of the dissolvent must be adjusted accordingly.

8. Collagenase/dispase solution for enzymatic harvesting of organoids (Stock: 100 mg/mL, working solution: 1 mg/mL).
  a. Dissolve 500 mg of collagenase/dispase powder in 5 mL dH_2_O.
  b. Mix by pipetting up and down.
  c. Prepare aliquots of 1 mL each and store at −20°C.
  d. To prepare working solutions, thaw one aliquot and dilute the stock solution in a 1:100 ratio in standard DMEM/F12, GlutaMAX.
  e. Filter the solution through a 0.22 μm filter.
  f. Always prepare working solution freshly.
  g. After defrosting, stock solution may be stored at 4°C for up to 4 weeks.
9. Supplemented Advanced DMEM/F-12.
  a. Prepare 485 mL of Advanced DMEM/F-12 by removing 15 mL of Advanced DMEM/F-12 from a 500 mL medium bottle in a laminar flow bench.
  b. Add 5 mL of penicillin/streptomycin (1% P/S, equal to a final concentration of 100 IU/mL of penicillin and 100 μg/mL of streptomycin) to the medium.
  c. Add 5 mL of 100× GlutaMAX supplement (200 mM) to the solution.
  d. Add 5 mL of 1 M sterile HEPES to the medium.
  e. Store at 4°C for up to 3 months.
10. Collagenase-II solution.
  a. Dissolve 1 g of collagenase-II in 50 mL of supplemented Advanced DMEM/F-12 to prepare a 20 mg/mL stock solution.
  b. Filter collagenase-II solution (20 mg/mL) sterile through a 0.22-μm filter.
  c. Store the collagenase-II stock solution aliquoted in dark reaction tubes at −20°C for up to 3 months.
  d. Prepare working solution by diluting the stock solution 1:5 for a final concentration of 4 mg/mL collagenase-II in standard (not Advanced) DMEM/F-12, GlutaMAX supplemented with 10 μM Y-27632.

***Note:*** Avoid repeated freeze-thaw cycles for the stock solution. Working solution can be stored for up to one week at 4°C.

11. Neutralization solution for collagenase-II solution (1% bovine serum albumin (BSA), 1% P/S in standard DMEM/F-12, GlutaMAX).
  a. Add 5 g of fatty acid free BSA to 495 mL DMEM/F-12.
  b. Add 5 mL of P/S (final concentration: 100 IU/mL penicillin, 100 μg/mL streptomycin) to the solution.
  c. Mix to completely dissolve the BSA.
  d. Filter the solution sterile through a 0.22-μm filter.
  e. Store the solution for up to 6 months at 4°C.
12. WNT3A-conditioned medium.
  a. WNT3A conditioned medium was prepared as described in the manufacturer`s protocol https://www.atcc.org/products/crl-2647 with slight modifications.
  b. Aliquot filtered medium and store at −80°C for up to 12 months.
13. RSPOI-conditioned medium.
  a. RSPO-conditioned medium was prepared as described in detail in the manufacturer`s protocol https://www.bio-techne.com/p/cell-culture/cultrex-ha-r-spondin1-fc-293t-cells_3710-001-01#product-datasheets with slight modifications.
  b. After selection, cells were seeded in 5 standard T175 cell culture flasks.
  c. When cells reached confluency, they were washed twice with 25 mL DPBS.
  d. 50 mL Supplemented Advanced DMEM/F-12 were added.
  e. After one week, medium was collected and transferred to a 50 mL conical tube.
  f. Centrifguation was performed 3,000 g for 8 min.
  g. Filter supernatant with a 0.2 μm filter.
  h. Store medium at −80°C for up to 12 months.

## Key resources table

**Table:** 

REAGENT or RESOURCE	SOURCE	IDENTIFIER
**Antibodies**		

Mouse monoclonal anti-human cytokeratin 19 (1:100)	Dako/Agilent	Cat.: M0888, RRID: AB_2234418
Mouse monoclonal anti-human nucleoli (1:200)	Abcam	Cat.: ab190710
Mouse monoclonal anti-Ki67(1:200)	Dako/Agilent	Cat.: M7240, RRID: AB_2142367
APC mouse anti-human HLA-ABC (1:5)	BD Transduction Laboratories	Cat.: 555555; RRID: AB_398603

**Biological samples**		

Porcine urinary bladder	Local Slaughterhouse of Ulm, Ulmer Fleisch GmbH, 89079 Ulm, Germany	N/A

**Chemicals, peptides, and recombinant proteins**		

A83-01	Tocris	Cat.: 2939/10
Accutase	Merck	Cat.: A6964
Advanced DMEM/F-12	Life Technologies	Cat.: 12634028
Agarose	Sigma-Aldrich	Cat.: A9539
Antibiotic-Antimycotic solution (100×)	Gibco	Cat.: 15240062
B27 supplement, without vitamin A	Thermo Fisher Scientific	Cat.: 12587-010
Calcium acetate hydrate	Sigma-Aldrich	Cat.: C1000, CAS: 114460-21-8
Collagenase/Dispase	Roche	Cat.: 11097113001
Collagenase-II	Gibco	Cat.: 17101015
Dispase-II	Sigma-Aldrich	Cat.: D4693, CAS: 42613-33-2
DMEM/F-12, GlutaMAX	Gibco	Cat.: 10565018
DPBS with MgCl_2_ and CaCl_2_	Sigma-Aldrich	Cat.: D8662
Human EGF	Peprotech	Cat.: AF-100-15
Fatty acid free BSA	Proliant	Cat# 68700, CAS: 9048-46-8
FBS Supreme, South America origin, fetal bovine serum, 0.2-μm sterile filtered	PAN Biotech	Cat.: P30-3031
Human FGF-10	Peprotech	Cat.: 100-26
Formaldehyde solution 3.5%–3.7%	Otto Fischar GmbH & Co. KG	Cat.: 27244
GlutaMAX^TM^ Supplement	Gibco	Cat.: 35050061
Human Gastrin I	Tocris	Cat.: 3006
HEPES solution, 1 M	Sigma-Aldrich	Cat.: H0887, CAS: 7365-45-9
Hydrochloric acid, 37%	Merck	Cat.: 30721-M
Insulin-Tansferrin-Selenium-X (ITS-X), 100×	Life Technologies	Cat.: 51500056
L-ascorbic acid	Sigma-Aldrich	Cat.: A4544
Corning® Matrigel® Growth Factor Reduced (GFR) Basement Membrane Matrix	Corning	Cat.: 354230
MCDB131	Invitrogen	Cat.:10372-019
Murine Noggin	Peprotech	Cat.: 250-38
N-acetylcysteine	Sigma-Aldrich	Cat.: A9165
Nicotinamide	Sigma-Aldrich	Cat.: N0636
Penicillin/Streptomycin (P/S)	Merck	Cat.: P4333
Peroxy-acetic-acid (PAA), 35wt.% sol. in diluted acetic acid, stabilized	Acros Organics	Cat.: 257750250, CAS: 79-21-0
Primocin	Invivogen	Cat.: ant-pm-2
Roti®-CELL Hanks’ BSS (HBSS)	Carl Roth	Cat.: 9122.1
R-spondin-1-conditioned medium	Prepared from 293T-HA-Rspo1-Fc Cells	N/A
Sodium acetate (NaCH_3_COO)	Carl Roth	Cat.: X891.2, CAS 127-09-3
Sodium bicarbonate	Sigma-Aldrich	Cat.: S5761
Sodium hydroxide (NaOH)	AppliChem	AP131687.1211
Y-27632 (2HCl)	Stem Cell Technologies	Cat.: 72307
WNT3A- conditioned medium	Prepared from L Wnt-3A (ATCC® CRL-2647™) with a G418(Gentamicin)-selectable marker	N/A

**Critical commercial assays**		

GeneJet RNA Purification Kit	Thermo Fisher Scientific	Cat.: K0732
Animal Tissue Genomic DNA Purification Mini Prep Kit	Genaxxon	Cat.: S5378.0050

**Experimental models: Cell lines**		

Human: HUES8 hESC line (NIH approval number: NIHhESC-10-0021) (recommended: <60 passages)	HSCI iPS Core, Harvard University, Cambridge, MA, USA	hES Cell Line: HUES8, RRID: CVCL_B207
HUES8-KRAS^G12D^ Cell Line (recommended: <60 passages)	Breunig et al.^2^	N/A
L Wnt-3A (ATCC® CRL-2647™) with a G418(Gentamicin)-selectable marker (recommended: <5 passages after selection)	ATCC®	Cat.: CRL-2647™
HA-R-Spondin1-Fc 293T Cells (recommended: <5 passages after selection)	Trevigen/Bio-Techne	Cat.: 3710-001-1
Pancreatic cancer patient-derived organoids (recommended: <10 passages)	This paper, Beutel et al.^4^	N/A
Pancreatic stellate cells (recommended: <30 passages)	Matthias Löhr,^9^	N/A

**Oligonucleotides**		

HMBS	Qiagen	QT00014462
GAPDH	Biomers	fwd: 5′- caggagcgagatccct-3′, rev: 5′-ggtgctaagcagttggt-3′
KRT-19	Biomers	fwd: 5′-ctacagccactactacacgac-3′, rev: 5′-cagagcctgttccgtctcaaa-3′
COL4A1	Qiagen	QT00005250
CDH1	Qiagen	QT00080143
PDX1	Qiagen	QT00201859

**Software and algorithms**		

Adobe Illustrator	Adobe	Creative Cloud
AxioVision software	Zeiss	https://www.micro-shop.zeiss.com/de/de/system/software+axiovision-axiovision+basissoftware-axiovision+software/10221/
Fiji	Schindelin et al.^10^	https://imagej.net/Fiji
GraphPad Prism 9	GraphPad Software, San Diego, California USA	https://www.graphpad.com
ZEN 3.1 imaging software (blue edition)	Zeiss	N/A
FlowJo v10	FlowJo, BD	https://www.flowjo.com/solutions/flowjo

**Other**		

Serrated forceps, various sizes	N/A	N/A
Forceps with hook, various sizes	N/A	N/A
Scissors, various sizes	N/A	N/A
Cell strainer, 40 μm, Nylon	Corning	Cat.: 352340
Filter for syringes, 0.2 μm	Sarstedt	Cat.: 83.1826.102
PCR tubes (Multiply®-Pro cup 0.2 mL, PP)	Sarstedt	Cat.: 72.737.002
CO2-resistant shaker	Thermo Fisher Scientific	Cat.: 88881101
Cell culture dish, 145/20 mm	Greiner Bio-One	Cat.: 639160
Cell culture dish, 6-well multiwell plate	Greiner Bio-One	Cat.: 657160

## Materials and equipment

**Table:** 10 mM sodium acetate and 5 mM calcium acetate

Component	Amount	Concentration
Sodium acetate (molecular weight 82.03 g/mol)	820.3 mg	10 mM
Calcium acetate hydrate (molecular weight 158.17 g/mol)	790.85 mg	5 mM
dH_2_O	800 mL	N/A
Sodium hydroxide/NaOH (1 M)	Variable, to pH 7.5	N/A
Hydrochloric acid/HCl (37%)	Variable, to pH 7.5	N/A
dH_2_O	To 1 L	N/A

Filter solution sterile before using. Store solution at room temperature for up to 1 year.

**Table:** Dispase-II-stock/working solution

Component	Amount	Concentration
**For stock solution:**		

10 mM sodium acetate and 5 mM calcium acetate	10 mL	N/A
Dispase-II powder	1 g	100 mg/mL

**Add for working solution:**		

DPBS with MgCl_2_ and CaCl_2_	90 mL	N/A

Filter solution sterile before using. Store stock solution at 4°C for up to 3 months. Prepare working solution freshly.

**Table:** Collagenase/dispase stock/working solution

Component	Amount	Concentration
**For stock solution:**		

dH_2_O	5 mL	N/A
Collagenase/dispase powder	500 mg	100 mg/mL

**Add for working solution:**		

DMEM/F12, GlutaMAX	45 mL	N/A

Filter solution sterile before using. Store stock solution at 4°C for up to 1 month. Prepare working solution freshly.

**Table:** Supplemented Advanced DMEM/F-12 for preparing the collagenase-II solution and conditioned medium

Component	Amount	Concentration
Advanced DMEM/F-12	485 mL	N/A
Penicillin/Streptomycin	5 mL	1% (equal to 100 IU/mL penicillin, 100 μg/mL streptomycin)
GlutaMAX, 200 mM	5 mL	2 mM
HEPES, 1 M	5 mL	10 mM

Prepare solution in a sterile setting and mix the solution. Store solution at 4°C for up to 3 months.

**Table:** Collagenase-II stock/working solution

Component	Amount	Concentration
**For stock solution:**		

Supplemented Advanced DMEM/F-12	50 mL	N/A
Collagenase-II powder	1 g	20 mg/mL

**Add for working solution:**		

DMEM/F12, GlutaMAX	200 mL	N/A
Y-27632, 10 mM	250 μL	10 μM

Filter solution sterile before using. Store stock solution at −20°C for up to 3 months. Store working solution at 4°C for up to 1 week. Smaller amounts of working solution can be prepared if sufficient for the respective experiment.

**Table:** Neutralization solution

Component	Amount	Concentration
DMEM/F-12, GlutaMAX	495 mL	N/A
fatty acid free BSA	5 g	1%
Penicillin/Streptomycin (10,000 IU/mL penicillin, 10,000 μg/mL streptomycin)	5 mL	100 IU/mL penicillin, 100 μg/mL streptomycin

Filter solution sterile before using. Store solution at 4°C for up to 6 months.

**Table:** Basal medium for culturing pluripotent stem cell-derived pancreatic duct-like organoids on PUB (see also Breunig et al.^2^^,^^3^)

Component	Amount	Concentration
MCDB131	955 mL	N/A
Fatty-acid-free BSA	20 g	2%
Sodium bicarbonate	1.754 g	1.754 g/L
Glucose	0.44 g	0.44 g/L
GlutaMAX	10 mL	200 mM Stock/ 2 mM final
L-ascorbic acid	44 mg	44 mg/mL
ITS-X	5 mL	0.5%
Antibiotic/Antimycotic	30 mL	3%

Mix the solution on a magnetic stirrer until all compounds are fully dissolved (min. 40 min). Protect from light. Filter the solution through a 0.22 μm filter. Prepare 50 mL aliquots and store medium at 4°C for up to 1 month.

**CRITICAL:** Using reagent grade, fatty-acid-free BSA is crucial. Batch-to-batch variation of BSA may impact the results of differentiation. Therefore, each batch should be tested and concentration in the basal medium may need to be adjusted.

**Table:** Medium for culturing patient-derived organoids (see also Beutel et al.^4^)

Component	Amount	Concentration
Advanced DMEM/F-12	35.69 mL	35.69%
HEPES, 1 M	365 μL	3.65 mM
GlutaMAX, 200 mM	365 μL	0.73 mM
Primocin, 50 mg/mL	200 μL	100 μg/mL
WNT3A-conditioned medium	50 mL	50%
R-spondin1-conditioned medium	10 mL	10%
B27 supplement, without retinoic acid, 50× stock solution	2 mL	2%
Nicotinamide, 1 M	1 mL	10 mM
N-acetylcysteine, 500 mM	250 μL	1.25 mM
Murine Noggin, 100 μg/mL	100 μL	100 ng/mL
Human EGF, 500 μg/mL	10 μL	50 ng/mL
Human FGF-10, 1 mg/mL	10 μL	100 ng/mL
Human Gastrin I, 100 μM	10 μL	10 nM
A83-01, 25 mM	2 μL	500 nM

Mix the solution. Protect from light. Filter the solution through a 0.22 μm filter. Prepare 50 mL aliquots and store medium at 4°C for up to 2 weeks.

### Boundary rings for seeding cells on the PUB

• Prepare rings for seeding cells by cutting 0.2-mL PCR-tubes to rings with a height of approximately 3 mm and a diameter of around 5 mm.
• Autoclave (121°C 20 min) rings before placing on PUBs.

***Note:*** Equivalent rings can be produced from different types of PCR tubes.

## Step-by-step method details

### Part I: Preparation of porcine urinary bladders for organ culture

**Timing: Alternative 1 – total timing: 2–3 days, hands-on-time: 2–3 h**

**Timing: Alternative 2 – total timing: 3–6 days, hands-on-time: 2–3 h**

**Timing: 30 min (for step 1)**

**Timing: 10 min (for steps 2a–j)**

**Timing: 15 min to 3 h (variable) (for steps 2k–m)**

**Timing: 5 min (for step 3a)**

**Timing: overnight (16–20 h) (for step 3b)**

**Timing: 10 min (for steps 3c–h)**

**Timing: 40 min per bladder (for steps 4a–d)**

**Timing: 24 h–48 h (for step 4e)**

**Timing: 30 min (for steps 4f–h)**

**Timing: 15 min (for steps 5a–b)**

**Timing: 24 h–48 h (for steps 5c–d)**

**Timing: 45 min (for steps 5e–j)**

**Timing: 24 h–72 h (for steps 5k–l)**

In a first step, PUBs were washed and de-epithelialized to enable the subsequent culture of engrafted pancreatic cells. De-epithelialization was achieved by enzymatic and mechanical treatment of the PUBs. PUBs were then either sterilized for long-term storage in PBS at 4°C or were immediately used.

**CRITICAL:** The use of sterile (autoclaved) forceps, scissors, and beakers at any time is mandatory to avoid handling-associated microbial contamination.

1. Obtaining porcine urinary bladders.
  a. Collect fresh porcine urinary bladders from the local slaughter-house within 3 h after isolation. Transportation on wet ice is recommended, however not obligatory.
  b. Ensure that PUBs are not frozen before processing.

***Note:*** PUBs should be processed as soon as possible after isolation to reduce microbial growth.

**CRITICAL:** Frozen PUBs should be discarded as scaffold integrity cannot be guaranteed.

**Figure 1:**
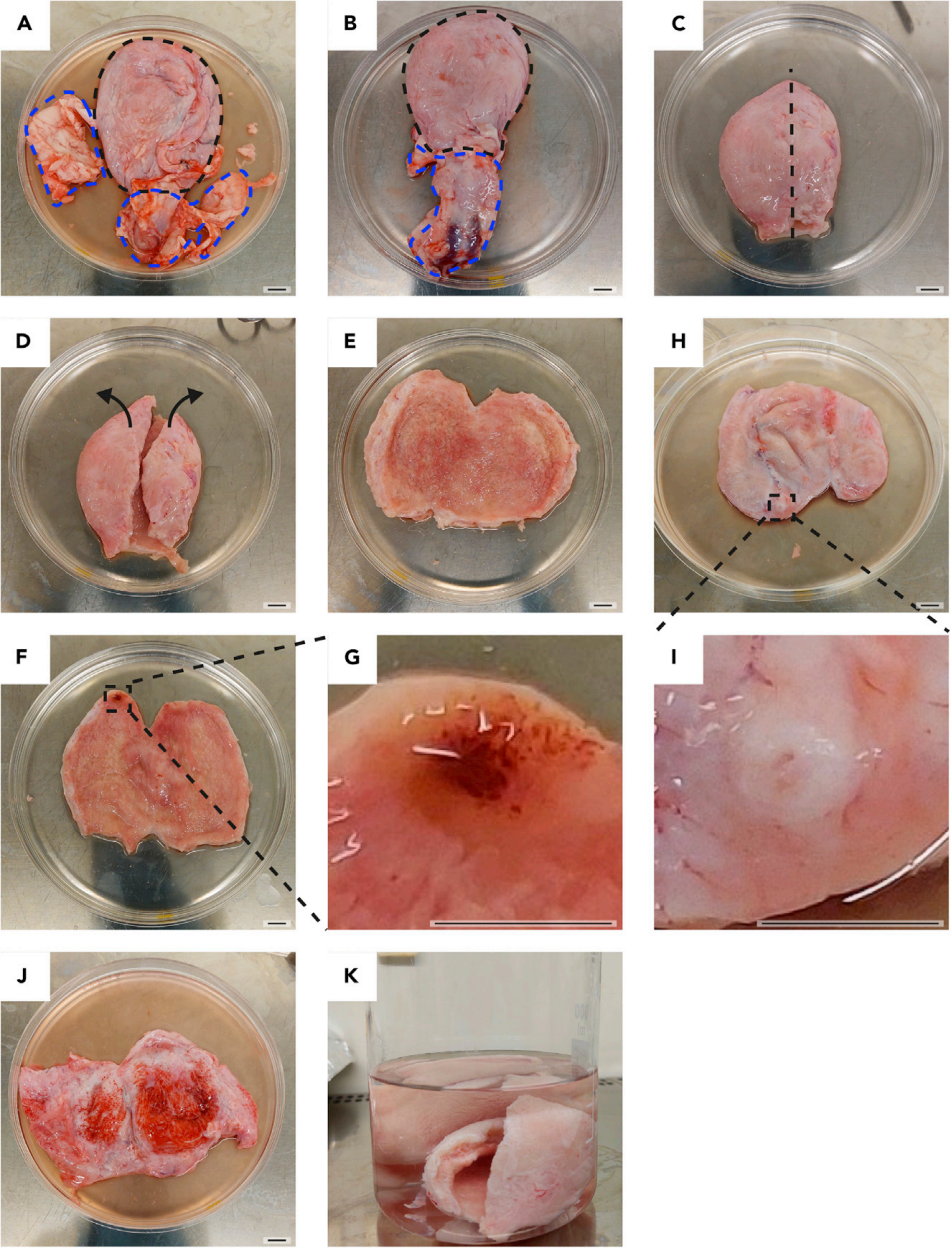
Cleaning of porcine urinary bladders (A) PUB in correct orientation in a 15-cm cell culture dish. Urinary bladder is marked in black. Adjacent tissue is marked in blue. (B) Urinary bladder after removal of adjacent fat tissue. Urinary bladder is marked in black. Urethra is marked in blue. (C) Urinary bladder after removal of fat tissue and urethra. The black line indicates opening of the bladder along an imaginary middle line vertical to the urethra. (D) PUB after sectioning along such middle line. Arrows indicate the direction to flap the two parts for complete opening. (E) Urinary bladder from the inner side displaying the urothelial layer. (F) Example of a small bleeding within the urinary bladder. After removal of the site of bleeding, the bladder can be still used. (G) Zoom-in to the small bleeding area presented in (F). (H) Urinary bladder after turning it upside down now displaying the outer wall. The square indicates one ureteral ostium which should be removed. (I) Zoom-in to the ureteral ostium. (J) Example of a large bleeding area on the outer side of the urinary bladder. The whole bladder should be discarded. (K) Example of a completely submerged bladder in wash solution. Scale bars indicate 1 cm.

**Figure 2:**
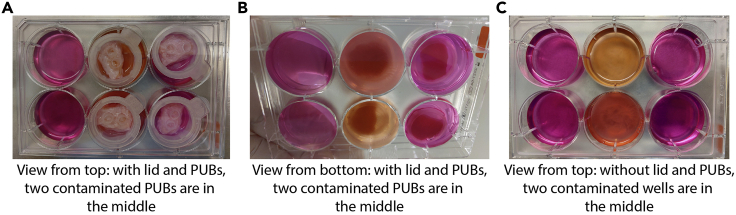
Example of a microbial-contaminated PUB (A) 6-well plate with two microbial contaminated PUBs in the two middle wells depicted from the top view. (B) View from below of the two contaminated wells in the middle. (C) View from top on the contaminated wells without the PUBs.

2. Cleaning of PUBs (Figure 1).
  a. Transfer PUB inside the laminar flow benches. Ensure the handling of PUBs under strict sterile conditions.
  b. Using sterile forceps, transfer PUBs from the transportation bag to a 15-cm cell culture dish, filled with DPBS.
  c. Adjust orientation of the PUB so that the urethra faces the operator (Figures 1A and 1B).
  d. Remove any adjacent tissue including fat, urethra, penis, vagina, or ureter (Figures 1A and 1B).
  e. Transfer the PUB into a new 15-cm dish, filled with DPBS (Figures 1B and 1C).
  f. After removal of the urethra, grab the bladder neck with forceps and use scissors to open it along the middle line (Figure 1C).
  g. Unfold the bladder and discard any remaining urine by washing the PUB in DPBS (Figures 1D and 1E).
  h. Perform a detailed visual inspection and check for bleedings, signs of infection, tumors, or other abnormalities (Figures 1E–1G).
    ***Note:*** Small bleedings (Figures 1F and 1G) can be trimmed away. In case of other abnormalities, discard the bladder. When signs of pus or infection are evident within the bladder, immediately discard the PUB, remove any contaminated tools, and rigorously clean the laminar flow bench before processing fresh bladders.
  i. Turn the bladder upside down and inspect the outer wall of the PUB for the same criteria as the inner side (Figures 1H–1J).
    ***Note:*** An example of a gross bleeding is presented in Figure 1J. Such PUB should be discarded.
  j. Remove the ureteral ostium (Figures 1H and 1I).
  k. Transfer the PUB into a beaker with excessive volume of bladder wash solution to ensure a complete submerging of the PUBs in the solution (Figure 1K).
  l. Incubate for at least 5 min at room temperature and transfer to a new beaker with bladder wash solution.
  m. Repeat this step three times.
    ***Note:*** It is crucially important to completely cover the PUBs with wash solution. Extending the washing steps to up to 60 min/step was tested and may result in fewer microbial contaminations.
3. De-epithelialization of PUBs (Methods video S1),
  a. Transfer PUBs to a beaker filled with the dispase-II working solution.
    ***Note:*** It is important to ensure permanent contact of dispase-II working solution with the urothelial layer of the PUBs. The urothelial layer is located on the inner, highly folded side of the PUB. We typically use around 10 mL dispase-II working solution per bladder.
  b. Incubate the PUBs overnight at 4°C.
  c. Transfer one PUB at a time to a new 15-cm cell culture dish filled with DPBS.
  d. Lift the PUB with forceps above the cell culture dish.
  e. Strip off the epithelial cell layer which was loosened by dispase-II treatment by using a second pair of forceps.
  f. Repeat this step from different angles of the bladder until complete removal of the epithelial layer.
    ***Note:*** Make sure to keep the PUB sterile while stripping off the urothelial layer. Take long sterile forceps (>15 cm) to avoid any direct contact between the PUB and the operator’s fingers.
    **CRITICAL:** Sterility is crucial to ensure smooth experimental procedure.
  g. Transfer de-epithelialized PUBs to a beaker filled with DPBS and sway PUBs to remove any remaining urothelial cells.
  h. Repeat step g in a new beaker with fresh DPBS.
    Troubleshooting 1.
4. Alternative 1: Taking PUBs directly into culture.
  ***Note:*** After cleaning and de-epithelization, PUBs can be taken directly into culture. Especially if interactions with the host, e.g., paracrine signaling, cell-cell communication with porcine cells etc., are of interest, the cultivation of non-sterilized PUBs may be beneficial. Good results for pancreatic cell culture on the PUB have been achieved by this method (please refer to Melzer et al.^1^). However, PUBs are degrading without additional PAA sterilization thereby limiting the culture period to a maximum of four weeks. Also, dropouts due to microbial contamination can be prevented by additional sterilization.
  ***Note:*** Always use sterile instruments to handle the PUBs.
  a. Transfer one PUB at a time into a new 15-cm cell culture dish filled with PBS.
  b. Cut the PUB into pieces of approximately 2 cm^2^ with sterile scissors.
  c. Add 6 mL of HBSS to each well of a 6-well plate.
    ***Note:*** To investigate potential contaminations, no antibiotics or antimycotics are added to the medium during this step. However, during cultivation of cells, media are supplemented with 3% antibiotic-antimycotic solution.
    ***Note:*** To reduce media to 3 mL per well of a 6-well plate, wells can be precoated with 3 mL of a 2% Agarose/PBS solution (sterilized by autoclavation). After solidification of the Agarose, medium can be filled up to 6 mL and cell strainers can be placed on top as described below. Agarose-filled plates can be also prepared in advance and stored for around two weeks at 4°C.
  d. Place one 40-μm cell strainer into one well of the 6-well plate. Ensure that the cell strainer is being laid up the rim of the well and the mesh on the bottom is in contact with the medium.
    ***Note:*** Only the lower part of the cell strainer should be in contact with the medium. The strainer must not be placed completely into the medium and formation of air bubbles below the cell strainer should be avoided. Depending on the manufacturer of the plates and cell strainers, the volume of HBSS per well might need to be adjusted to fulfill these requirements. Be aware that not all manufacturers provide cell strainers that are suitable for this kind of application.
  e. Place a piece of PUB into each cell strainer of the 6-well plates. Ensure that the former urothelial side (tunica mucosa) is directed toward the air-phase (up) and the outer wall (tunica serosa) faces the medium (down). Incubate in a cell culture incubator for 24 h at 37°C with a 5% CO_2_ and 21% O_2_ atmosphere.
    ***Note:*** This period can be extended to 48 h, which might ease the detection of microbial contamination. However, non-sterilized bladders will lose viability and start to degrade. Therefore, longer pre-incubation times are not recommended for the direct culture method.
  f. Check for any signs of microbial contamination by visual and microscopical inspection of the wells. Proceed with the next steps in case of no sign of contamination.
    ***Note:*** Contamination-free setting is critical for further experiments. Macroscopic signs of infections include cloudy medium and significant color changes of the medium. However, microbial contamination may be more discrete and should be also carefully investigated with a microscope. An example of contaminated and non-contaminated PUBs is presented in Figure 2.
  g. Prepare new 6-well plates containing 6 mL of the respective cell culture medium, including 3% antibiotic-antimycotic solution and in case of PSC-derived cultures 10 μM Y-27632.
    ***Note:*** A variety of different media, cytokines, and other substances were used in previous PUB experiments. While none of the conditions had an obvious impact on the PUB itself (concerning morphology of the scaffold), pre-testing of specific media formulations should be considered for new conditions and cell types.
    ***Note:*** To enhance cell viability of pluripotent stem cell-derived pancreatic cell types, add 10 μM Y-27632 for cell seeding.
  h. Transfer non-contaminated PUBs with their respective cell strainers into new 6-well plates by using sterile forceps, always ensuring that no air bubbles are present below the nylon meshes and PUBs are not covered by medium but only in contact on the lower site.
    Troubleshooting 2.
    Troubleshooting 3.
5. Alternative 2: Sterilization of PUBs.
  ***Note:*** Follow these steps directly after step 3 as an alternative to step 4. Sterilization of PUBs is achieved by treatment with peroxy-acetic acid as initially described in Rosario et al.^11^
  a. Prepare a beaker with around 350 mL sterilization solution.
    ***Note:*** Ensure coverage of all PUBs with sterilization solution. Typically, we use 350 mL sterilization solution for a maximum of 5 bladders.
    **CRITICAL:** Sterilization with PAA reduces the likelihood of PUB-derived contamination. However, extreme precaution must be maintained when handling the PUBs to prevent operator-derived contamination.
  b. Transfer PUBs from DPBS to the sterilization solution with sterile forceps.
  c. Incubate the PUBs in sterilization solution for 24 h at 4°C–8°C.
  d. Repeat the sterilization for another 24 h with new sterilization solution.
  e. Wash PUBs twice with DPBS for at least 5 min.
    ***Note:*** After washing, PUBs can be stored in DPBS at 4°C–8°C for up to 3 months. Longer storage periods were not tested.
    **Pause point:** At this point, PUBs can be stored at 4°C–8°C in DPBS before continuing the procedure.
    Troubleshooting 4.
  f. Transfer one PUB at a time to a 15-cm cell culture dish filled with DPBS.
  g. Cut the PUB into pieces of approximately 2 cm^2^ with sterile scissors.
  h. Prepare 6-well plates containing 6 mL of the respective cell culture medium, including 3% antibiotic-antimycotic solution and in case of PSC-derived cultures also 10 μM Y-27632.
    ***Note:*** See part I-4c for the use of 3 mL 2% Agarose/PBS-coated plates to reduce culture media.
  i. Place a cell strainer into each well as indicated in step 4.
  j. Transfer PUB pieces into cell strainers, ensuring that the former urothelial side is facing the air and the tunica serosa is in contact with the medium.
  k. Incubate in a cell culture incubator for 24–72 h at 37°C.
    ***Note:*** The medium may rapidly turn yellow due to sudden pH change which is caused by the ongoing PAA-evaporation in case of little residual PAA. The yellow medium color switch alone should not be considered as a sign of contamination as long as no other signs are present. Carefully assess bacterial contamination under the microscope and continue with uncontaminated bladders.
    ***Note:*** No decline of quality was observed between 24–72 h, but 72 h is recommended in order to allow exclusion of contaminated bladders. Longer periods were not tested but are likely possible. However, when working with temperature-sensitive media or supplements such as cytokines that are degraded over time, a media change may be necessary before proceeding with the cell seeding to avoid loss of cell viability or differentiation status.
  l. Check for any signs of contamination before proceeding with the cell seeding.
    ***Note:*** Although sterilization has been performed, microbial contamination cannot be totally excluded. Handling of the PUBs is challenging and comprises many steps prone to contamination. However, according to our experience, the rate of microbial contaminations drops to less than 5% of the PUBs if handled properly. Examples of contaminated PUBs are depicted in Figure 2.
    **CRITICAL:** Take quality control samples for histological examination of the de-epithelialization. Samples should include a native PUB control and a de-epithelialized PUB control after dispase-II treatment and also after PAA-treatment. Antibodies specific for the markers of your cells of interest should be used to discriminate them from porcine cells in case the de-epithelialization is not complete.
    Always use sterilized tools and work under a sterile laminar flow hood to handle PUBs.

Methods video S1. Step 3: De-epithelialization of the porcine urinary bladder Two pairs of forceps are used to remove the urothelial cell layer after enzymatic overnight digestion. One pair of forceps is used to hold the urinary bladder, the other is used to strip off the epithelial cell layer.

### Part II: Cell seeding and on-PUB culture

**Timing: Total timing: 4–5 h, hands-on-time: 2–3 h, for ongoing culture: 15 min**

**Timing: 30 s per PUB (for steps 6a–b)**

**Timing: 2 h-variable (for step 6c)**

**Timing: variable (for step 7a)**

**Timing: 45 min (for steps 7b–i)**

**Timing: 15 min (for step 8)**

**Timing: 15 min (for step 9)**

Prior to cell seeding, small rings will be placed on the PUBs serving as a boundary during seeding and subsequent culture. The herein described workflow worked robustly for different cell lines and cell numbers and across single cells, whole and fragmented organoids as indicated in Table 1. However, the optimal number of cells should be adjusted according to particular applications.

6. Preparing bladders for seeding of cells.
  a. Place two rings on each bladder using sterile forceps.
    ***Note:*** Rings should be placed straightly on PUBs to prevent cell suspension spilling during seeding.
    ***Note:*** We typically use two rings on each bladder, however, depending on applications and available cell numbers one might prefer the use of a single ring per bladder.
  b. Gently press the ring into the scaffold to ensure close contact of the ring with the PUB.
  c. Wait for at least 2 h before seeding cells.
    ***Note:*** Incubation period is flexible and can be extended to several days if PUBs are prepared in advance. However, a minimum amount of time (2 h) is critical to ensure proper settling and connection to the bladder.
7. Seeding of cells.
  a. Harvest the cells/organoids following standard protocols for each specific cell/organoid type. A detailed description for pancreatic progenitor cells and organoids is provided in Breunig et al.^3^
    ***Note:*** We typically harvest organoids by digestion of the Matrigel with 1 mg/mL collagenase/dispase for 2–4 h at 37°C in a cell culture incubator with 5% CO_2_ (typically 500 μL per well of a 24-well plate). Using this kind of enzymatic digestion, we have observed a proper digestion of the Matrigel without a significant decline in cell viability after harvesting. We stop the reaction with neutralization solution (twice the amount of the collagenase/dispase) and centrifuge the organoids for 5 min at 200 g before proceeding with the next steps. If singularization of cells is necessary, we wash the organoids once in DPBS and resuspend them in accutase, before incubating them 30 min at 37°C in a waterbath. After 15 min and at the end of the incubation, we gently pipette the organoids up and down to enhance the enzymatic digestion. The reaction is stopped by adding twice the amount of neutralization solution and centrifugation for 5 min at 200 g before proceeding with the next step.
  b. Count cell/organoid number. Cell/organoid numbers of different pancreatic cell types and co-cultures that worked well in our hands as well as their tested cultivation periods are provided in Table 1. Adjust cell/organoid numbers according to the number of intended rings.
    ***Note:*** Cultivation periods can be adjusted for specific purposes, e.g., time course of tumor development, invasion, maturation of pluripotent stem cell-derived pancreatic progenitors etc. So far, we did not culture cells longer than 4 weeks on non-sterilized bladders and did not culture cells longer than 8 weeks on sterilized bladders. While degradation of non-sterilized bladders limits the maximal cultivation time to around 4 weeks, sterilized bladders are likely stable far beyond 8 weeks.
    ***Note:*** Although low cell/organoid numbers were tested and led to a successful engraftment, the histological search for a small engraftment can be cumbersome. Hence, we advise adhering to recommended cell numbers giving rise to large and easily identifiable engraftments.
  c. If co-cultures are intended, mix different cell types well by pipetting.
    ***Note:*** The exact number and ratio of cells for co-culture experiments should be optimized for the specific application but the recommended numbers in Table 1 can serve as a starting point. Coculture approaches have proven to be a very useful tool to study, e.g., cell crosstalk between epithelial and stromal cells and to investigate stromal redrafting by tumor cells. Cell numbers should be adjusted to recommended cell numbers as initial starting point (recommended starting point 2,000 organoids per ring, 166,000 single cancer cells per ring, and respective amounts of stromal cells). However, testing of cellular composition is necessary to find the optimal ratio and number of each cell type for specific co-culture approaches of interest.
  d. Centrifuge cell suspensions and remove the supernatant completely.
  e. Resuspend cells/organoids in the respective cell culture medium with a volume of 15 μL medium per ring. In case of PSC-derived cell cultures, add 10 μM Y-27632 to the medium.
    ***Note:*** The seeding volume was optimized for our system including organoids with a maximum organoid size of around 150 μm. Bigger/smaller organoids may need adjustments for the organoid numbers or seeding volume. However, we do not recommend to significantly increase the seeding volume as organoids will be mostly located then in the fragile Matrigel/medium layer rather than in contact with the PUB.
    ***Note:*** Using the same media formulation in *ex vivo* PUB experiments as in the *in vitro* culture of the cell type of interest, is a good starting point and led to robust and good results in our experiments. However, we observed that on-PUB propagation allowed removal of certain supplements such as FBS, cytokines, e.g., EGF, FGF-10 etc. Examples are given in Table 2. However, head-to-head comparisons should be performed for the specific application before depleting any supplements from the original *in vitro* cell culture medium.
  f. For every ring, add 15 μL GFR-Matrigel to the cell suspension to reach a final concentration of 50% GFR-Matrigel.
  g. Add 30 μL of cell suspension to each ring.
  h. Carefully check for any potential spilling outside the rings and mark respective rings on the cell culture plates.
    ***Note:*** The likelihood of successful engraftment is very low if cell suspension spills outside of the ring.
    ***Note:*** Seeding without Matrigel was tested successfully for murine cancer cells.
  i. Place the cell culture plates in a cell culture incubator at 37°C until the next medium change.
    Troubleshooting 4.
8. Ongoing culture.
  a. Once to twice a week, prepare new 6-well plates containing 6 mL of the respective culture medium for the cell types seeded on the PUBs.
    ***Note:*** Frequency of medium changes depends on the presence of heat-labile molecules, e.g., cytokines, in your medium. If heat-labile molecules are within the medium, more frequent media changes might be necessary for your application.
    ***Note:*** See Part I/4c for the use of 3 mL 2% Agarose/PBS-coated plates to reduce culture media.
  b. Transfer PUBs from the old plate to the new plate using sterile forceps.
9. Treatment of PUBs with drugs or small molecules to investigate cytotoxicity and transgene expression.
  ***Note:*** PUB can be used to investigate the effect of distinct compounds on the cellular behavior. For example, doxycycline can be added to the culture medium to modulate transgene expression with Tet-on systems (reviewed in.^13^). In our case, treatment of PUBs with the same doxycycline concentrations (3 μg/mL) as used in the *in vitro* cell culture counterparts resulted in robust transgene (HA-tagged KRAS^G12D^-IRES-mCherry) expression.^1^ In addition, the investigation of cytotoxic components and their effect on cancer cells or patient-derived organoids in this organ culture model is feasible. In our setting, we have tested the effects of olaparib (3.7 μM) and irinotecan (1.1 μM).^1^
  While doxycycline, olaparib and irinotecan have been added to the medium below the PUB requiring a penetration of the drug through the PUB scaffold, a direct treatment of cells can be also achieved by drug addition to the boundary rings. Thus, the use of other drugs and the duration of their application must be tested in respect to the specific application of interest as not all drugs might properly penetrate through the PUB scaffold.
  a. Add the respective drug or small molecule in the concentration of interest to the medium.
  b. Perform medium change as described in Part II-3a-b.
  c. Alternatively, add the drugs in the desired concentration in a small volume, e.g., 30 μL, to the rings.
    ***Note:*** Treatment can also be directly started by adding the respective drug to the medium during seeding of the cells.
    **CRITICAL:** Always use sterile forceps for handling PUBs.

**Table 1:** Tested and recommended cell numbers and cultivation time of different pancreatic cell types

Cell type	Cell/organoid number (per ring)		Maximum tested cultivation time
	Tested range	Recommended range/number	
PSC-derived pancreatic progenitor cells (single cells)	300,000–500,000 single cells	300,000–500,000 single cells	8 weeks
PSC-derived pancreatic duct-like organoids (fragmented organoids)	1,000–2,500 organoids (corresponding to approximately 100,000–250,000 cells)	>2,000 organoids	4 weeks
Patient-derived organoids (fragmented organoids)	300–20,000 organoids (corresponding to approximately 30,000–2,000,000 cells)	>2,000 organoids	4.5 weeks
Pancreatic stellate cells (single cells)	30,000–2,000,000 single cells	500,000 single cells	3 weeks
Murine pancreatic cancer cells (single cells)	83,000–1,250,000 single cells	>166,000 single cells	6 weeks
PBMCs (only in co-cultivation)	30,000–250,000	N/A	5 days
	**Ratio**		
Patient-derived organoids : pancreatic stellate cells	1:100 (whole organoids : single cells)	application-dependent	10 days
Patient-derived organoids : pancreatic stellate cells : PBMCs	1:15:50 (whole organoids : single cells : single cells)	application-dependent	5 days
Murine cancer cells : pancreatic stellate cells	1:2–1:3 (single cells : single cells)	application-dependent	3 weeks

**Table 2:** Medium formulation *in vitro* and on PUB

Cell type	Culture medium *in vitro*	Alternative culture medium formulation on PUB
Pancreatic progenitors	For ductal differentiation: basal medium as in Breunig et al.^2^^,^^3^ and Merkle et al.^12^ + 50 ng/mL EGF, 50 ng/mL FGF-10, 10 mM nicotinamide, 10 μM ZnSO_4_, 50 nM MSC2530818, 50 ng/mL KGF, 10 μM Y-27632	Basal medium as in Breunig et al.^2^^,^^3^ and Merkle et al.^12^ + 5% FBS
Pancreatic duct-like organoids	Basal medium as in Breunig et al.^2^^,^^3^ and Merkle et al.^12^ + 50 ng/mL EGF, 50 ng/mL FGF-10, 10 mM nicotinamide, 10 μM ZnSO_4_	Basal medium as in Breunig et al.^2^^,^^3^ and Merkle et al.^12^
Patient-derived organoids	Organoid medium as in Beutel et al.^4^	DMEM/F-12 + 10% FBS
Murine cancer cells, pancreatic stellate cells	DMEM/F-12 + 10% FBS	DMEM/F-12

### Part III: Histological examination of PUBs and harvesting of samples for RNA/DNA/FACS analysis

**Timing: Histology - Total timing: 2 days, hands-on-time: 15 min to variable**

**Timing: RNA/DNA/FACS – Total timing: 5–8 h**

**Timing: 5 min (for steps 10a–c)**

**Timing: overnight (for step 10d)**

**Timing: 5 min (for steps 10e–g)**

**Timing: 14 h/variable (for step 10h)**

**Timing: 5 min (for steps 10i–j)**

**Timing: variable (for step 10k)**

**Timing: 10 min (for steps 11a–f)**

**Timing: 60 min/variable (for steps 11g–i)**

**Timing: 20 min (for steps 11j–o)**

**Timing: 30–45 min (for steps 11p–q)**

**Timing: 20 min (for steps 11r–u)**

**Timing: variable (for step 11v)**

In this step, the harvesting of *ex vivo* on-PUB cultures for histological analysis is described in detail. For histological analysis, the orientation of the sample and the discrimination between porcine cells and human/murine cells is critical.

**Figure 3:**
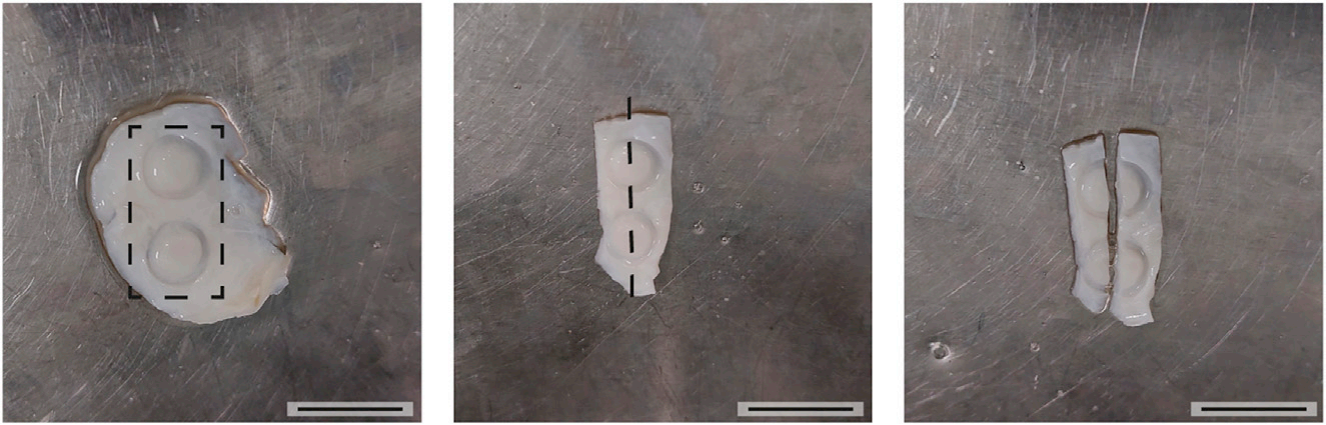
Preparation of fixed PUB samples for histological analysis Tissue outside of the implantation site can be removed (indicated by rectangle). If PUBs are used to investigate invasion they should be divided in the middle of the ring (indicated by the dashed line). The image on the right presents an example of a PUB after cutting the middle of the implantation site for vertical sectioning. Scale bars indicate 1 cm.
  g. Perform a standard automated dehydration series.
  h. To embed the PUBs into paraffin, place the bladder upper side down (180° inversion).
    ***Note:*** Embedding and sectioning the PUBs with this horizontal orientation will lead to a plethora of structures. Also, structural arrangement of the engraftment can be investigated with this approach. For examples see Melzer et al.,^1^. However, invasion should only be carefully investigated on sections obtained from samples with this orientation, no truthful vertical interaction of the cells with the scaffold is mirrored. Examples are depicted in Figure 4.
    ***Optional:*** To investigate the process of invasion, turn the two halves obtained from cutting the middle of the implantation site of one ring (in g) by 90° so that the sectioned side of the PUB faces the bottom during paraffin embedding.
    ***Note:*** This vertical orientation will enable investigation of the interaction between the engrafted cells and the PUB scaffold. This enables evaluation of invasion and migration toward the former subepithelial stroma and muscle layer of the scaffold. While this is an excellent technique to analyze the cells with strong connection to the PUB scaffold, cells that are still located in the former Matrigel layer may be lost during dehydration or handling procedures. Hence, the number of detectable cells may be less than after horizontal sectioning.

10. PUB processing to formalin-fixed paraffin-embedded tissue (FFPE).
  a. Fill the wells of a new 6-well plate with 3.7% formaldehyde.
    ***Note:*** Formaldehyde is toxic and hazardous. Follow the manufacturer`s indications on how to handle and store it. Wear personal protection equipment (gloves, clothing, eye, and face protection) and avoid exposure to it.
  b. Take PUB pieces out of the cell strainers and transfer each to one well filled with formaldehyde.
  c. Drown the PUBs very carefully in the formaldehyde solution and ensure complete coverage.
    ***Note:*** At this stage, rings should not be removed by force from the PUBs if they do not come off by themselves. Please ensure that no air bubbles are within the rings when covered by formaldehyde. Do not add the formaldehyde on top of the PUBs to avoid mechanical removal of cells. Be especially careful when working with samples from short-term experiments. The shorter the experiment, the more Matrigel will be present, and cells will be suspended in the Matrigel layer which is more sensitive to mechanical forces.
  d. Fix the cells overnight at 4°C–8°C.
  e. Take the PUBs out of the formaldehyde and carefully remove the rings to avoid the detachment of adherent cells.
  f. Remove any tissue distant to the rings from the PUBs with scissors or a scalpel (Figure 3).
    ***Optional:*** Cut the piece of PUB in the middle of the former ring area, in case vertical sectioning and investigation of invasion into the former sub-epithelial stroma or muscle layer of the PUB is desired (Figure 3).
    ***Note:*** Be careful when cutting into the middle of the implantation site as the seeded cells, especially in short-term condition, might be otherwise removed from the scaffold.

**Figure 4:**
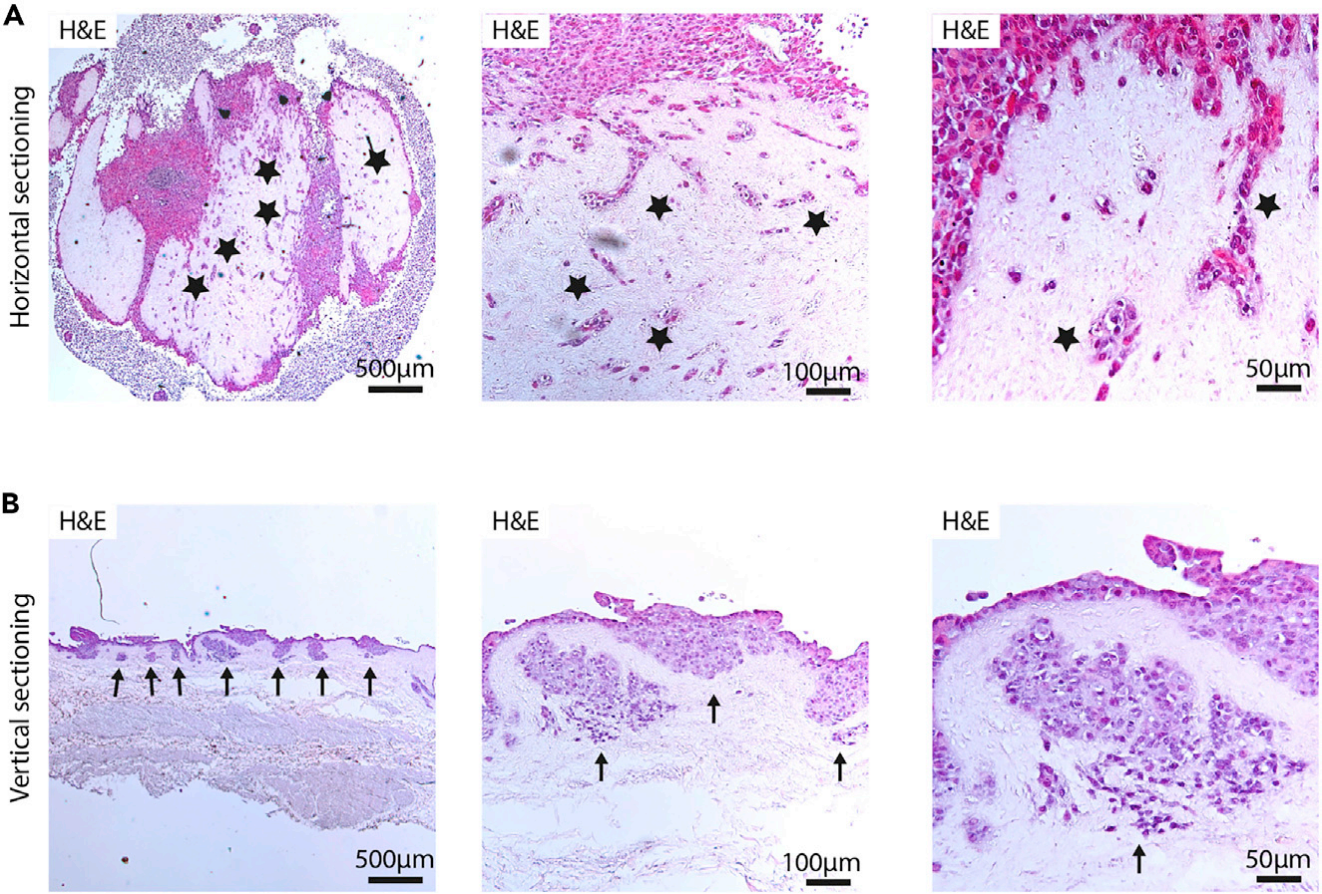
Examples of successful engraftment and sectioning orientation (A) Example of horizontal sectioning of a tumor cell engraftment after 21 days. Dispersed tumor cells are found within the scaffold indicative of potential invasion. Stars indicate examples of suspected invasive cancer cells. However, invasion in this orientation should be carefully considered. (B) Example of tumor cells invading the PUB as investigated by vertical sectioning. Arrows indicate tumor cell infiltration.
  i. Perform standard histological staining procedures and confirm identity of your cells by specific staining.
    ***Note:*** Especially when working with non-sterilized PUBs, it becomes difficult to distinguish in H&E staining between urothelial cells of the pig and the cells of interest in case of incomplete removal of epithelial cells. The use of human-specific antibodies such as anti-human nucleoli, anti-human cytokeratine 19, as well as human specific Ki-67 (see also key resources table, and^1^ supplemental information), however, enables a clear discrimination between human and porcine cells. No issues in distinguishing human or murine cells from other porcine cells after treating PUBs with PAA during the initial preparation arose during our investigations.
    Troubleshooting 1.
    Troubleshooting 4.
    **CRITICAL:** Careful handling is necessary to avoid removal of cells by mechanical forces. “Invasive” cells may also be observed when investigating sections prepared parallel to the surface (horizontal orientation). However, such observation should be regarded carefully as numerous foldings of the inner bladder wall may lead to false positive results if not sectioned vertically.
11. Harvesting cells for RNA-, DNA-, and flow cytometry -based assays (Figure 5).
  a. Fill the wells of a 6-well plate with DPBS.
  b. Transfer PUB pieces from cell strainers to the 6-well plate.
  c. Carefully submerge the PUBs in the DPBS and shake the plate to enable proper washing.
  d. Remove the rings from the PUBs very carefully.
    ***Note:*** Especially in short-term conditions, cells may be located in the Matrigel/medium layer rather than being fully attached to the scaffold. This may lead to a certain degree of instability with the subsequent risk of losing cells of interest when removing the rings from the scaffold. In that case, try to capture those Matrigel-embedded cell clusters with a pipette and further process them along with the PUBs in subsequent steps.
  e. Prepare a new 12-well plate. Fill the wells with 2 mL of collagenase-II working solution.
    ***Note:*** Other enzymatic digestion approaches may also work. In short-term experiments with cultivation of cells for up to 5 days, we achieved comparable RNA yield with either accutase (30–45 min incubation) only and or collagenase/dispase (2 h incubation) digestion only. However, in long-term conditions (>2 weeks), we observed a much better detachment of cells when using the collagenase-II solution for isolation of cells from the PUB scaffold.
    ***Note:*** Also porcine cells of the scaffold PUB can be isolated by this method, which can become relevant for certain downstream applications.
  f. Take PUBs from the DPBS and place them with the cell containing side up into the collagenase-II working solution.
    ***Note:*** Ensure complete coverage of the PUBs with the collagenase-II sollution.
  g. Incubate for 60 min at 37°C on an orbital shaker with 115 rotations per minute.
    ***Note:*** Incubation on an orbital shaker outperformed the enzymatic digestion without a shaker.
  h. Shake the plates carefully to allow cells to spread throughout the whole well.
  i. Check under the microscope if cells are floating around. In case of very few detached cells, prolong the incubation in the collagenase-II solution to up to 180 min.
    ***Note:*** We did not observe a decline in cell viability, however, cell viability should be assessed for individual cell lines, e.g., in flow cytometry (Figure 6).
  j. Use forceps to lift the PUBs and hold it against the edges of the well. Aspirate the collagenase-II solution containing the cells with a pipette and rinse it against the former ring area of the PUBs to loosen any remaining cells.
  k. Transfer collagenase-II solution to a 15-mL centrifugation tube.
  l. Use 4 mL neutralization solution to wash the wells and after rinsing the bladders again, transfer the cell suspension to the same tube.
  m. Perform a centrifugation at 200–250 × *g* for 5 min.
  n. Aspirate supernatant and resuspend in 2 mL DPBS.
  o. Repeat the centrifugation at 200–250 × *g* and aspirate the DPBS.
    ***Note:*** Cells are now ready for DNA- or RNA-isolation. We applied the GeneJet RNA Purification Kit (Thermo Fisher, https://www.thermofisher.com/document-connect/document-connect.html?url=https://assets.thermofisher.com/TFS-Assets%2FLSG%2Fmanuals%2FMAN0012664_GeneJET_RNA_Purification_UG.pdf) or the Animal Tissue Genomic DNA Purification Mini Prep Kit (Genaxxon, https://www.genaxxon.com/shop-all-products/rna-dna-protein-reinigung/dna-reinigungskits/554/tissue-genomic-dna-purification-mini-spin-column-kit-50-columns?number=) according to manufacturer`s instructions, respectively. However, to perform flow cytometry, cells must be singularized as indicated in the following steps.
    ***Note:*** As the collagenase-II digestion leads to isolation of porcine cells as well, precautions have to be taken into consideration for further downstream analysis. There is a high chance of porcine DNA-contamination. RNA is likely degraded over time and during treatment with peroxy-acetic acid. However, even in sterilized samples, remnant porcine nucleic acid fragments including RNA were detected in scaffold-only samples. Hence, choosing human-specific (or murine-specific) primers is mandatory to avoid measuring porcine scaffold gene expression. Indeed, after reverse transcription and performing a qRT-PCR analysis, we hardly detected any mRNA gene expression in PAA-treated scaffold-only samples for several different primers. Examples of DNA-, RNA- concentration and gene expression of two housekeeping genes (HMBS, GAPDH) as well as four other genes (KRT19, CDH1, PDX1, COL4A1) are depicted in Figure 7.
    Troubleshooting 5.
  p. Resuspend cell pellet in 1 mL accutase.
  q. Incubate at 37°C in a waterbath for 30 min. Mix well by pipetting up and down after 15 min.
    ***Note:*** Incubation can be extended to 40 min if cell clusters are still present in microscopic examination.
  r. Take cells from the waterbath and pipette up and down again until cells are homogeneously resuspended using a 1,000 μL pipette and respective tips.
  s. Add 2 mL of neutralization solution and mix well.
  t. Perform a centrifugation at 200–250 × *g* for 5 min.
  u. Aspirate supernatant and resuspend in 2 mL DPBS.
  v. Subject the cells from the PUBs to standard flow cytometry staining and measurement.
    ***Note:*** When subjecting cells to flow cytometry analysis, antibody specificity for human/murine cells should be tested. Representative flow cytometry results for cell viability and the expression of the pan-human marker HLA-ABC are demonstrated in Figure 6.

**Figure 5:**
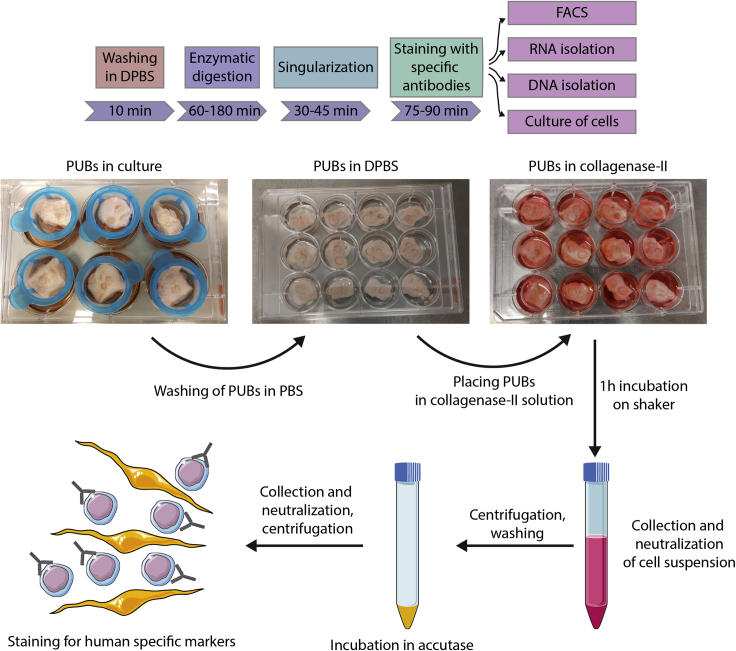
Workflow for isolation of cells from PUBs The upper panel is a schematic representation of the workflow and timing necessary to isolate cells from the PUBs. The middle panel shows the transfer of PUBs from cell strainers to 12-well plates filled with DPBS. Tissue not adjacent to the rings is removed with scissors. After shaking PUBs in DPBS and careful removal of the rings, PUBs are transferred into new wells, filled with collagenase-II working solution. After a 1–3 h incubation on a shaker, the supernatant, containing the cells of interest, is collected in a centrifugation tube, PUBs are additionally rinsed with neutralization solution, and neutralization solution is added to the centrifugation tube. After centrifugation and washing in DPBS, cells are incubated in accutase for 30 min to achieve singularization of the cells. PUB-derived cells are then stained with specific antibodies to perform flow cytometry.

**Figure 6:**
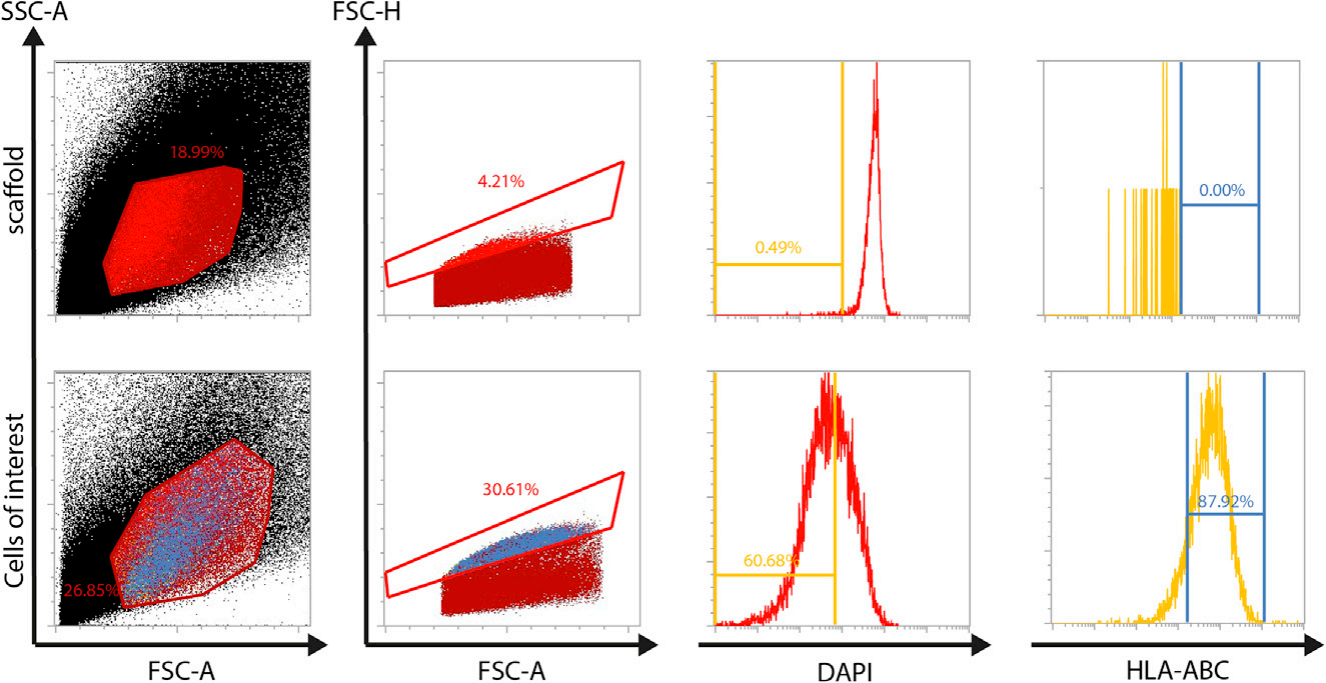
Representative flow cytometry results after isolation of cells from the PUB PUBs without prior cell seeding (scaffold) and PUBs with seeding of human pancreatic progenitor cells and cultivation for 6 weeks were treated with collagenase-II. Subsequent singularization was performed with accutase. Cells were stained for DAPI to exclude dead cells and with a human-specific HLA-ABC-APC antibody according to manufacturer`s recommendations. Presence of viable cells expressing HLA-ABC was only detected in samples with prior seeding of human cells.

**Figure 7:**
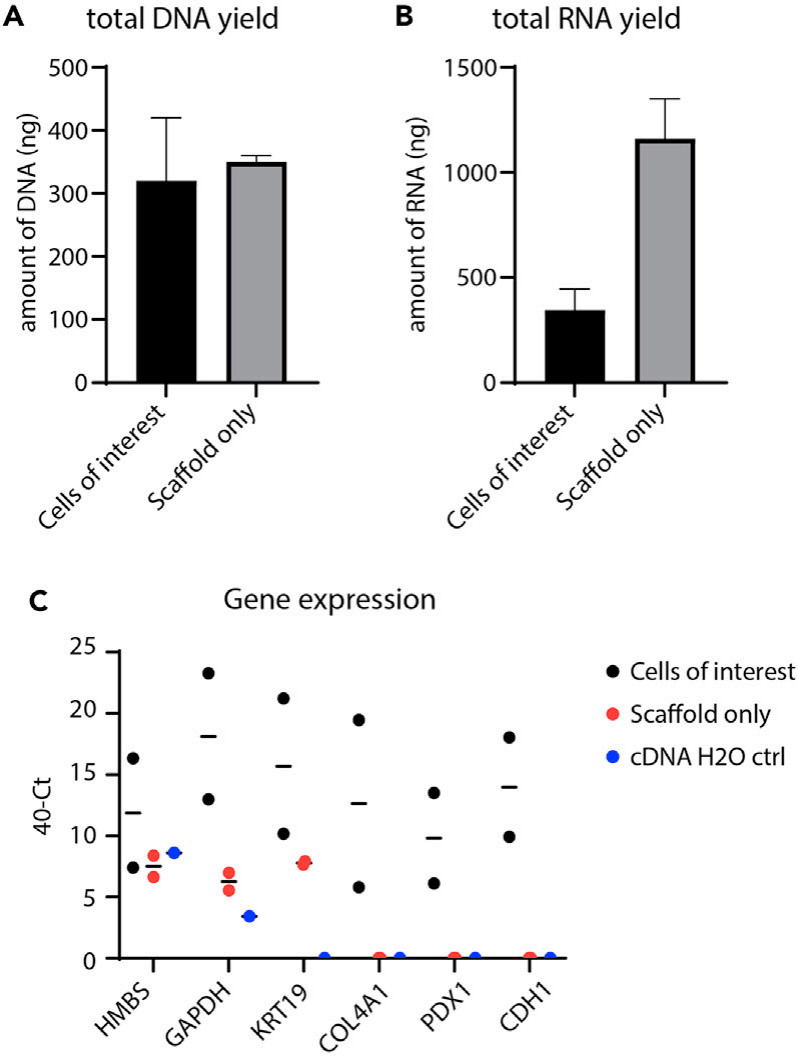
Representative results of DNA and RNA yields after isolation of cells from PUB (A) Bar graphs of total DNA yield after digestion of PUBs with cells of interest and scaffold only samples as measured with a Nanodrop. Mean ± S.E.M. (B) Bar graphs of total RNA yields of PUBs with cells of interest and scaffold only samples as measured with a Nanodrop. Of note, the Nanodrop device does not differ between fragmented and non-fragmented nucleic acids. Mean ± S.E.M. (C) Gene expression is displayed as 40-Ct-values of cells harvested from PUBs (Cells of interest vs. scaffold only vs. cDNA H2O control sample). If threshold was not reached Ct-values were set to 40. Line indicates mean. n = 2 independent PUBs.

## Expected outcomes

The most powerful feature of the *ex vivo* PUB model is the formation of tissue-like structures from a variety of different pancreatic cell systems. Hence, histological analysis including immunohistochemical and immunofluorescence staining allows a detailed analysis of cellular processes such as differentiation, atypia, dysplasia, invasiveness, and cellular interactions. In this respect, the PUB model did not only serve as scaffold for developmental maturation of PSC-based differentiations but also for the study of oncogenic transformation capacity. Examples for histological readouts of cultures on non-sterilized and sterilized PUBs are presented in Melzer et al.^1^ as well as in Figure 4. The histological readout proved to be highly reliable if precautions are taken and cells are not spilled during seeding.

Flow cytometry-based readouts can identify the target population when a broadly expressed marker such as HLA-ABC is chosen (Figure 6). From around 300,000 pancreatic progenitor cells that were seeded per ring in two rings per bladder, between 5,000–20,000 human, living cells were detected in flow cytometry analysis. In addition, around 500 ng total DNA per bladder could be retrieved (Figure 7). RNA isolation typically yielded between 250 ng and 400 ng in total per bladder.

## Limitations

The major drawback of using non-sterilized urinary bladders is the potential risk of microbial contamination. Together with ongoing degradation limiting the use of non-sterilized PUBs to 3–4 weeks, we strongly recommend implementing PAA sterilization, if the intended application does not require interaction with host cells. After sterilization, the contamination rate was below 5% and long-term culture for at least 8 weeks was possible (no longer timepoint was investigated but seems likely to be feasible). While histological analysis of PUB samples works very robustly, the application of further downstream applications, e.g., flow cytometry -, RNA-, or DNA-analysis yields rather few cells and low amounts of nucleic acids requiring sensitive isolations kits. In addition, contamination with porcine nucleic acids makes it necessary to carefully design and choose human-/murine-specific primers. Although reproducibility is high for histological readouts, the yield and purity of RNA/DNA may vary from experiment to experiment. To overcome potential drawbacks and variance of DNA and RNA analysis, it is recommended to work at least in PUB triplicates (hence, typically 6 rings per condition). Also, the enrichment of cells of interest by sorting should be considered as option to overcome such hindrances.

Albeit we tested many different cell types and media formulations in the context of pancreatic development and carcinogenesis, the implementation of not yet tested cell systems might need additional optimization.

## Troubleshooting

### Problem 1

Incomplete de-epithelialization.

**Figure 8:**
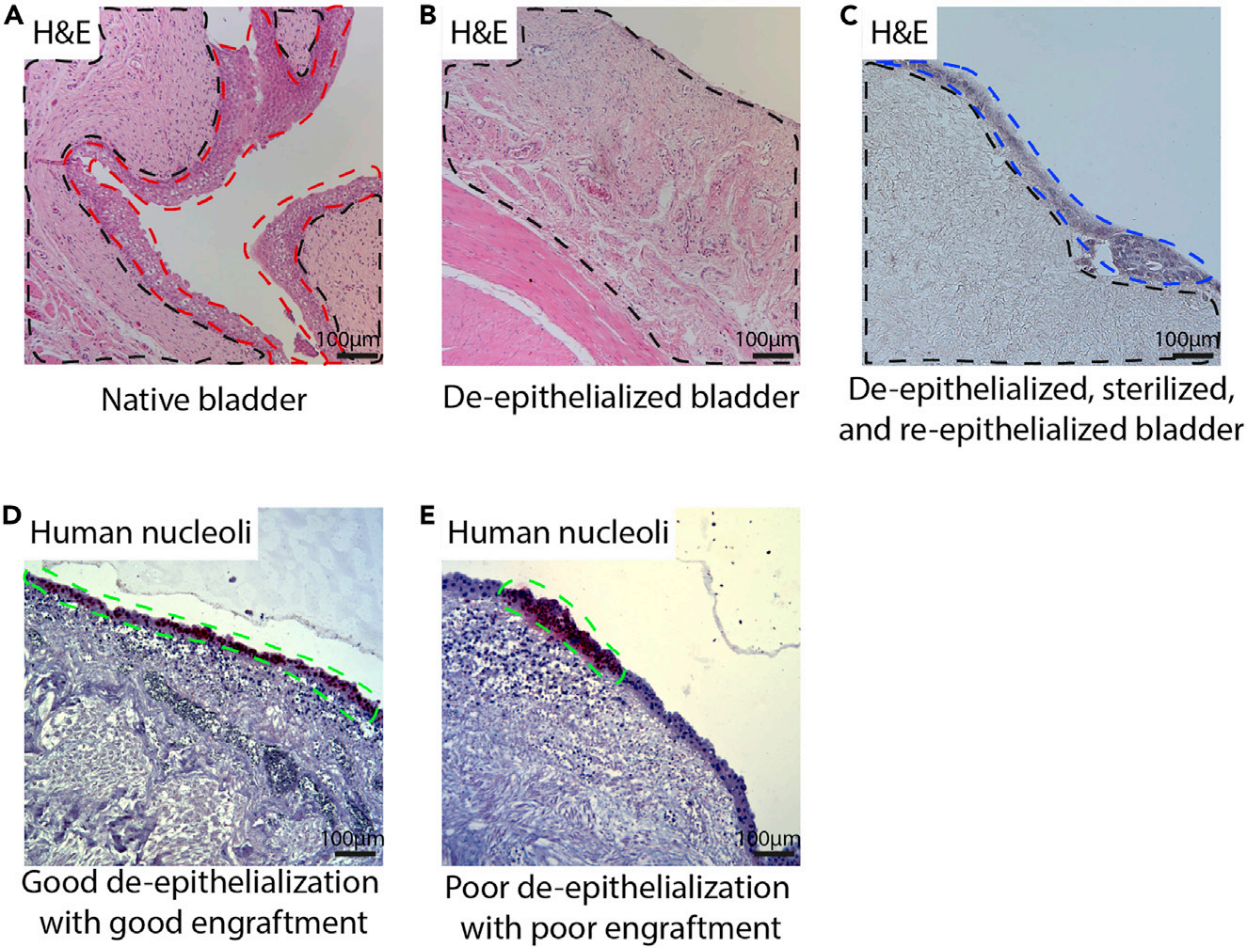
Examples for de-epithelialized and re-epithelialized PUBs (A) Example of a native porcine urinary bladder with intact urothelial cell layer (indicated by red line) and sub-epithelial stroma (indicated by black line). (B) Example of a successful de-epithelialization with no remaining urothelial cell layer. Stroma is indicated by the black line. (C) Example of a de-epithelialized, sterilized, and re-epithelialized urinary bladder. The black line indicates the stromal compartment where no or only few nuclei and intact cells are found. The blue line indicates the cells of interest that were seeded onto this scaffold 2 weeks before histological examination. (D) Example of a good engraftment after proper de-epithelialization on a non-sterilized urinary bladder. Human cells are stained by an anti-human nucleoli antibody (Abcam, ab190710) and are indicated by the green line. (E) Example of a poor engraftment of human cells on a non-properly de-epithelialized porcine urinary bladder. Human cells are stained by an anti-human nucleoli antibody and are indicated by the green line. Next to the human cells, remnants of the porcine urothelial cell layer are present. Scale bars indicate 100 μm.

During the preparation of the PUBs, incomplete de-epithelialization might occur (step 3). This will result in poor engraftment and difficulties to distinguish host cells from cells of interest. Examples for good and poor de-epithelialization and subsequent engraftments are displayed in Figure 8.

### Potential solution

• Prolong and deepen the mechanical stripping of the porcine urinary bladder as indicated in Methods video S1 (step 3).
• Consider treating the PUBs with PAA, as this results in a decline of viability and integrity of porcine cells leading to better de-epithelialization (Figure 8C) (step 5).
• Always use de-epithelialized control samples without additional cell implantation for histological examination on the quality of de-epithelialization.
• Use species-specific antibodies to identify your engraftment. Include native porcine urinary bladders as negative controls.

### Problem 2

Microbial contamination.

Microbial contamination can occur due to two main reasons. 1. Contamination can be carried along from the beginning, as PUBs are not harvested under sterile conditions (step 2). 2. Sterile handling of PUBs and trans-well systems is more difficult than typical *in vitro* cultures (step 8).

### Potential solution

• Contamination carried along from the beginning:
  • Carefully check freshly obtained bladders for any sign of contamination. In case of doubts, discard the bladder (step 2).
  • Consider treating PUBs with PAA to sterilize them. This step leads to a highly reduced rate of microbial contaminations. This is especially relevant, as bacterial infections might only present at later stages after reaching a certain detection limit despite being present from the beginning (step 5).
  • Increase antibiotic concentration in the medium. However, any influence on cell viability should be carefully controlled.
• Contamination during ongoing culture (step 8).
  • Always take sterile forceps and use a cleaned laminar flow bench for handling bladders.
  • Ensure that cell culture incubators are cleaned and sterilized regularly.
  • Transfer PUBs to new wells for each medium change.
  • Do not place forceps directly on the working area of laminar flows.

### Problem 3

Degradation of PUB.

The culture on non-sterilized bladders is limited to 3–4 weeks by the ongoing degradation of the PUB.

### Potential solution

• Perform a sterilization with PAA for long-term culture (step 5).
• Choose bladder pieces with a high vertical thickness. Usually, pieces will be around 3 mm thick. Choosing pieces with a vertical thickness of around 5 mm can thereby extend the cultivation for several additional days up to one week.

### Problem 4

Poor engraftment.

The engraftments are either 1. small, 2. not viable, or 3. hard to identify. Small engraftments might be caused by (1a) spilling of cells during seeding outside of the rings, (1b) starting with too few cells, (1c) or improper handling after fixation. Poor viability in the engraftment might be due to (2a) an undetected microbial contamination, (2b) a cell culture-specific problem such as media formulation or cellular stress during cell seeding, or (2c) the presence of relevant amounts of PAA in PUBs. And finally, problems with identification of the graft mostly arise because of (3a) incomplete de-epithelialization or (3b) loss of implanted cells during embedding and sectioning.

### Potential solution

• Small engraftments:
  ○ Consider increasing the number of seeded cells. We do not advise to increase the seeding volume, as changes in Matrigel thickness might affect its connection to the PUB scaffold (step 7).
  ○ Handle PUBs carefully after fixation for histological examination. Avoid detachment of any cells that are only loosely attached to the PUB (step 10).
  ○ In case of cell detachment, process also these cells together with PUBs.
  ○ Ensure that the rings for seeding are properly attached and pressed into the scaffold to avoid any spilling during the seeding process (step 6).
• Poor viability of engraftments:
  ○ Ensure that cells are handled correctly before seeding (cell-specific enzymatic digestion procedure, gentle pipetting, etc.). Check if cells are suitable to be seeded without addition of Y-27632. In case they are not, add Y-27632 during seeding and for the first medium change (step 7).
  ○ Discard any PUBs that show signs of contamination.
  ○ If PAA is used during preparation of the PUBs make sure that no PAA is left before cell seeding. Increase the amount and duration of washing rounds after treatment with PAA. Prepare PUBs up to 5 days in advance to allow for further evaporation of PAA (step 5).
  ○ Consider systematic medium comparisons for propagation of the intended cell type on-PUB (step 7).
• Difficulties in identifying engraftment (step 10).
  ○ If no cells have been identified, continue sectioning to exclude that the engraftments are out of the current sectioning area.
  ○ If cells are present but no clear engraftment is identified, use antibodies specific for your cells to exclude contamination with porcine urothelial cells.
  ○ Refer to problem 1 for a refinement of de-epithelialization.
  ○ Starting with horizontal sectioning is recommended, as this orientation typically contains more cellular structures than vertical sectioning. However, sectioning some PUBs vertically and others horizontally might complement each other for a better refined overall structure.

### Problem 5

Low DNA/RNA yield.

Concentration and/or quality of isolated DNA/RNA might be poor (step 11).

### Potential solution

• Use isolation methods/kits for nucleic acids that are designed for high yields with low input material.
• Include replicate samples processed in parallel for histology to assess the size of the engraftment and its cell viability.
• Consider pooling several bladders from the same condition for increasing the input material.

## Resource availability

### Lead contact

Further information and requests for resources and reagents should be directed to and will be fulfilled by the lead contact, Alexander Kleger, alexander.kleger@uni-ulm.de.

### Materials availability

This study did not generate new unique reagents.

## Data Availability

This study did not analyze/generate datasets/codes.
